# Validation of the Aging Perceptions Questionnaire Short on a sample of community-dwelling Turkish elderly migrants

**DOI:** 10.1186/s12955-017-0619-7

**Published:** 2017-02-21

**Authors:** Anne Slotman, Jane M Cramm, Anna P Nieboer

**Affiliations:** Department of Socio-Medical Sciences, Institute of Health Policy and Management, Erasmus University Rotterdam, P.O. Box 1738, 3000 DR Rotterdam, The Netherlands

**Keywords:** APQ-S, Aging perception, Validity, Reliability, Turkish migrant

## Abstract

**Background:**

Aging perceptions have been found to be major contributors to health in old age. To quantitatively explore aging perceptions among elderly Turkish migrants, valid and reliable Turkish-language instruments are needed. The objective of the current study was to examine the construct validity and reliability of the Turkish-language version of the seven-dimension Aging Perceptions Questionnaire Short version (APQ-S) in a sample of community-dwelling elderly Turkish migrants in the Netherlands.

**Methods:**

A questionnaire including the Turkish-language APQ-S was administered to 438 community-dwelling Turkish migrants aged 65–99 years who resided in Rotterdam, the Netherlands. The APQ-S includes 21 items in seven dimensions (*timeline chronic* and *cyclical*, *consequence positive* and *negative*, *control positive* and *negative*, and *emotional representations*). The questionnaire also contained items measuring well-being, physical and mental health-related quality of life, and number of chronic conditions.

**Results:**

The latent factor model of the Turkish APQ-S was found to have an acceptable fit (root mean square error of approximation = .06; standardized root mean square residual = .07; comparative fit index = .90). Each indicator loaded significantly on its corresponding latent factor, and standardized factor loadings > .40 supported the convergent validity of the Turkish APQ-S dimensions. The APQ-S was also found to have acceptable construct validity in terms of its inter-factor structure and its expected associations with various health measures and age, gender, educational level, and marital status. Contrary to expectations, income level was not associated with any APQ-S dimension. With the exception of *timeline cyclical* (*α* = .56), each APQ-S dimension had acceptable reliability, with Cronbach’s alpha values ranging from .75 (*timeline chronic*) to .88 (*control positive*). Most APQ-S dimension scores differed significantly between elderly Turkish migrants and a general population of elderly Rotterdam residents, with Turkish elders having more negative and less positive aging perceptions.

**Conclusion:**

The Turkish-language version of the APQ-S is a psychometrically valid and reliable instrument for the assessment of aging perceptions among elderly Turkish migrants. Further research is needed to gain insight into aging perceptions and their health and sociodemographic correlates in this population.

**Electronic supplementary material:**

The online version of this article (doi:10.1186/s12955-017-0619-7) contains supplementary material, which is available to authorized users.

## Background

Although it was thought that Turkish migrants, along with other groups of guest workers, would remigrate once their temporary contracts expired, a considerable number of these migrants eventually settled permanently in their respective host societies and are currently aging in countries to which they are not native [1]. Elderly Turkish migrants comprise one of the most vulnerable elderly groups in Western Europe [2]. As the majority of these migrants have received little or no schooling, have been recruited mainly for low-skilled and low-paid manual labor, and live on small, incomplete pensions, many are less financially equipped to support themselves in old age [2]. Besides financial hardship, factors such as language difficulties, poor housing conditions, unfamiliarity with services and amenities for elders, and the increasing intolerance toward Muslim migrants in Europe create a less favorable environment for aging [2]. This environment may impact the health and well-being of elderly Turkish migrants. Not surprisingly, compared with their native peers, more of these migrants have poor physical and mental health [3–6], worse physical health-related quality of life (HRQoL) [7], and more functional limitations [4, 8, 9] and chronic conditions [4]. In addition, they report being lonely more often [10]. This disadvantaged position in society may eventually negatively affect how these migrants experience and perceive their aging as a process and old age as a phase of life. Various studies have underscored the importance of aging perceptions, which have been related to a multitude of physical, psychological, and behavioral outcomes. For example, positive perceptions of aging have been associated with greater well-being [11, 12], functional health [12–14], and quality of life [15, 16]; less reported loneliness [17], anxiety [18], and depressive symptoms [14, 18, 19]; more health-promoting behaviors [20–23]; and increased life expectancy [24–26]. Furthermore, the possibility of altering maladaptive aging perceptions makes these perceptions an attractive target for interventions [27].

Despite its possible health impacts, research on aging perceptions among Turkish migrants, or migrants in general, is scarce [4, 28, 29]. Large-scale quantitative studies are needed to understand how elderly Turkish migrants experience and perceive their aging, and how these factors are related to their well-being and health in the context of their migrant status and the (socioeconomic) disadvantages it often implies.

Such research requires the use of instruments that can validly and reliably measure aging perceptions among elderly Turkish individuals. One quantitative instrument available for the assessment of aging perceptions is the Aging Perceptions Questionnaire Short version (APQ-S) [12], which is the 21-item version of the original 32-item APQ [14]. The lengthier APQ has been proven to be valuable for the assessment of aging perceptions [14]. The APQ-S is a valuable alternative to the lengthier APQ, which reduces the response burden among older adults. The APQ-S still represents the seven dimensions of aging perceptions. It is a multidimensional instrument based on the theoretical framework of Leventhal’s social regulation model [30], which holds that aging is a stressor, as it places specific demands on the individual. To make sense of their aging and behave accordingly, people form internal representations of the process [14]. These representations can be categorized using a series of dimensions, which correspond to the following (sub)dimensions of the APQ-S:

- **Timeline.** This dimension encompasses perceptions of aging that are *chronic* (constant awareness of aging, e.g., “I am always aware of my age”) or *cyclical* (fluctuating awareness of aging, e.g., “I go through phases of feeling old”). A chronic awareness of aging has been associated with physical inactivity [31] and poor health [32].
- **Consequences.** This dimension concerns beliefs about the impact of aging on various life domains, which can be *positive* (e.g., “As I get older I appreciate things more”) or *negative* (e.g., “Getting older restricts the things that I can do”). Positive beliefs about the impact of aging have been related to greater well-being [11], and negative beliefs have been linked to depression [19] and lesser well-being [11].
- **Control.** This dimension relates to beliefs about the extent to which one can control *positive* (e.g., “Whether I continue living life to the full depends on me”) and *negative* (e.g., “How mobile I am in later life is not up to me”) aspects of aging. Ample research has shown that a reduced sense of control in late life negatively influences various health-related outcomes (for an overview, see Lachman, Neupert, and Agrigoroaei [33]).
- **Emotional representations.** This dimension encompasses the negative emotional responses one has to aging (e.g., “I get depressed when I think about getting older”). Negative emotional responses, such as worries concerning one’s aging, have been related to poor physical and functional health [34], distress [35], and maladaptive coping strategies [36].

A previous study has supported he psychometric properties of the APQ-S [12]. However, whether this instrument can be used reliably and validly to assess aging perceptions among elderly Turkish migrants remains to be determined. Hence, the main objective of the current study was to examine the reliability of the Turkish-language version of the APQ-S, in terms of internal consistency and discriminant and convergent validity, using a sample of elderly community-dwelling Turkish migrants in the Netherlands.

## Methods

### Participants and procedure

Data from the first data collection period of the Healthy and Well in the Netherlands study were used for this study [37]. The ongoing Healthy and Well in the Netherlands study focuses on the health and well-being of Turkish, Moroccan, and Dutch community-dwelling elders residing in Rotterdam, the Netherlands. In the first data collection period (April–July 2015), a preliminary subsample of elderly Turkish migrants was recruited for assessment of the psychometric properties of the study instruments. All Turkish elders meeting the inclusion criteria, i.e., community-dwelling Rotterdam residents aged ≥ 65 years, were included in the sample. Participants were sent written invitations with questionnaires and self-addressed envelopes. Reminders were sent in cases of non-response, followed by at-home visits by Turkish interviewers. The written invitation, questionnaire, and reminder were provided in Turkish and Dutch. Prior to participation, each respondent was informed about the aims of the study and its anonymous and voluntary nature. Of 2350 Turkish elders approached, 106 were found to be ineligible due to serious medical conditions (e.g., dementia), nursing home residence, change of address, or death. Of the remaining sample, 438 elders participated in the first data collection period. Three hundred thirty-nine respondents refused to take part in the study. The remaining 1468 respondents could not be contacted and will be approached in the second data collection period. Hence, the preliminary response rate was 20%. Ten additional respondents were excluded due to submission of largely incomplete questionnaires, resulting in a final study sample of 428.

As part of the validation process, Turkish-language APQ-S results were compared with those from a Dutch-language APQ-S validation study conducted with a general sample of elderly community-dwelling Rotterdam residents [12]. Respondents in that study were sampled randomly and stratified by age group (70–74, 75–79, 80–84, and ≥85 years) and neighborhood, which was weighed proportionally according to the district population ratio. This population was older than the Turkish sample (≥70 vs. ≥65 years). The dataset used contained information for 1220 respondents aged 70–99 years (mean = 78.59 years, standard deviation [SD] = 6.17 years), of whom 57.6% were female and 15.2% were of non-Dutch origin (e.g., Turkey, Germany, Surinam, and Greece) (see Slotman and colleagues [12] for more detailed information on the dataset).

### Measures

#### Aging perceptions questionnaire short version

Ageing perceptions were measured using the 21-item Ageing Perceptions Questionnaire Short (APQ-S) [12]. The APQ-S assesses ageing perceptions across seven (sub)dimensions as described in the background. The (sub)dimensions are as follows: **Timeline –** A person’s awareness of ageing, which can be constant (*timeline chronic/acute*: e.g., “I always classify myself as old”) or vary through time (*timeline cyclical*: e.g., “I go through phases of feeling old”). **Consequence** – The believed impact of ageing on various life domains that can be positive (*consequence positive*: e.g., “As I get older I get wiser”) and/or negative (*consequence negative*: e.g., “As I get older I can take part in fewer activities”). **Control** – The perceived level of control one has over aspects related to their ageing. These ageing-related aspects can be positive (*control positive*: e.g., “Whether I continue living my life to the full depends on me”) and negative (*control negative*: e.g., “Slowing down with age is not something I can control”). **Emotional representations** – Negative emotional reactions one has towards their ageing (e.g., “I get depressed when I think about getting older”). Each dimension is comprised of three items and includes answer categories, ranging from 1 ‘totally disagree’ to 5 ‘totally agree’. The scale for control negative items is reversed, and responses were recoded so that higher scores were indicative of greater perceived negative control. Professional native Turkish translators living in the Netherlands translated the APQ-S items into Turkish (see Additional file 1). All of these translators were official and certified, and studied Turkology, ensuring high-quality translation. The procedure was as follows: a first translator translated the Dutch instrument into Turkish. A second translator then checked all steps of this procedure (e.g., spelling, grammar, terminology, and cultural interpretation of words). A third translator performed back translation to ensure that the items had been translated properly. Finally, the questionnaire was tested among older native Turkish people to ensure content validity for the target population.

#### Well-being

Well-being was measured using the Social Production Function Instrument for the Level of Well-being (SPF-IL) [38]. This 15-item instrument measures whether a person’s needs for stimulation, comfort, behavioral confirmation, affection, and status are met. Examples of items are “Do people help you if you have a problem?” and “Do you really enjoy your activities?”. Mean scores ranging from 1 (never) to 4 (always) were calculated, with higher scores indicating greater well-being. Data from participants who responded to fewer than 10 SPF-IL items (*n* = 3) were excluded from the analyses. Previous studies have supported the reliability and validity of the SPF-IL for use in elderly populations [39, 40]. In the current study, the SPF-IL had a Cronbach’s alpha value of .85, indicating good reliability.

#### Physical and mental health-related quality of life

The physical component summary scale (PCS) and the mental component summary scale (MCS) of the 12-item Short Form Health Survey (SF-12) were used to measure the physical and mental aspects of HRQoL, respectively [41]. The SF-12 is the short version of the well-known 36-item Short Form Heath Survey [42]. The PCS measures physical functioning, bodily pain, role limitations due to physical pain, and general health perceptions. Items measuring vitality, social functioning, role limitations due to emotional problems, and mental health comprise the MCS. Component scores ranging from 0 to 100 were calculated for each scale, with higher scores denoting better physical and mental HRQoL (see Ware, Kosinski, and Keller [43] for information concerning PCS and MCS scoring). Following the advised method [43], respondents with missing data on the PCS (*n* = 19) or MCS (*n* = 20) were excluded from the respective analyses. The PCS and the MCS were found to have acceptable psychometric properties in previous studies [41, 44, 45].

#### Number of chronic conditions

Respondents were asked to report the number of chronic conditions they had in the past 12 months. A list of 14 chronic conditions (e.g., cancer, depression) was provided, along with the option to list other conditions that were not among the selection. Only conditions that were classified as chronic by O'Halloran, Miller, and Britt [46] were added to the count.

#### Sociodemographic variables

Age, gender, monthly net income, educational level, and marital status were included as control variables. Monthly net income, educational level, and marital status were dichotomized. Male gender, monthly net income ≥ €1350, elementary schooling or higher, and married/living together functioned as reference categories. Sample characteristics are presented in Table 1.

**Table 1:** Descriptive statistics for variables used in the regression analyses

	*M* (SD)/%	Missing (%)
Well-being (1 – 4)	2.77 (.53)	.70
Physical HRQoL (1 – 100)	46.10 (11.47)	4.50
Mental HRQoL (1 – 100)	52.76 (17.60)	4.70
Nr of chronic conditions (1 – 10)	2.72 (1.87)	.00
Age (65 – 95)	72.82 (5.21)	.00
Gender (%)		
Female	45.26	.00
Income (%)		
< € 1350	68.01	13.51
Education (%)		
< Elementary	56.16	.71
Marital status (%)		
Single/widow	28.44	.47

*Note*. The displayed means and standard deviations or proportions are calculated after missing data was handled on the APQ-S items (*n* = 422)

### Analyses

Data were analyzed in the following sequence:

1. Raw data were assessed using SPSS 23.0 [47]. Items were inspected for floor and ceiling effects by calculating item means and SDs and visually inspecting histograms and Q-Q plots. Item non-response was examined using a cut-off point of 10%, above which non-response was seen as problematic.
2. The construct validity of the APQ-S was examined using structural equation modeling (SEM) with Mplus 7.31 [48]. Through confirmatory factory analysis (CFA), a measurement model was specified by loading each item onto its respective latent factor (i.e., the *timeline*, *consequence*, and *control* sub-dimensions and *emotional representations*). The fit of the resulting model was then evaluated using the chi-squared statistic in combination with the standardized root mean square residual (SRMR), root mean square error of approximation (RMSEA), and comparative fit index (CFI). A small and non-significant chi-squared statistic was seen as an indicator of exact model fit. However, due to its sensitivity to large samples, this statistic should be interpreted with caution [49]. Cut-off points of SRMR ≤ .08, RMSEA ≤ .06, and CFI ≥ .90 were used [50]. Missing values were handled using the full information maximum likelihood method, which methodologists generally favor for most CFA and SEM applications [49, 51, 52]. Data from participants who responded to fewer than 11 APQ-S items (*n* = 6) were excluded from analyses.
  The validity of the APQ-S, in terms of the hypothesized factor structure, was assessed by inspecting standardized and unstandardized factor loadings and modification indices (MIs). Strong (**λ** > .40) and significant positive factor loadings were seen as indicators of convergent validity [49, 53]. MIs > 10 were seen as indicators of areas of localized strain (i.e., correlated measurement errors due to, for example, similarity of item wording or item cross-loading [49]), and, thus, lack of validity of the internal structure of the APQ-S.
3. To further assess the validity of the APQ-S’s inter-factor structure (i.e., convergent validity), inter-factor correlations were calculated using SPSS 23.0 and compared with the correlations reported by Slotman and colleagues [12]. Correlations exceeding .80 were seen as evidence of a possible lack of discriminant validity [49].
4. Cronbach’s alpha values were calculated to examine the internal consistency of each (sub)scale. Values of 0.7 > α ≥ 0.6 were seen as indicating questionable consistency, those of 0.8 > α ≥ 0.7 were considered to be acceptable, 0.9 > α ≥ 0.8 was considered to be good, and values of α ≥ 0.9 were seen as reflecting excellent consistency [54].
5. Additional tests of construct validity were conducted by examining hypothesized associations between the APQ-S dimensions and other measures. First, the APQ-S dimensions were correlated with measures of well-being (SPF-IL), physical and mental HRQoL (PCS and MCS, respectively), and the number of chronic conditions. As previous studies have shown that more negative aging perceptions are related to worse well-being and health outcomes [11, 15, 16, 19], similar results were expected in this study. Specifically, we hypothesized that higher *timeline chronic* and *cyclical*, *consequence negative*, and *emotional representations* scores and lower *consequence positive* and *control positive* and *negative* scores would be associated with lower well-being and HRQoL scores and more chronic conditions.
  Discriminant validity was examined by testing for differences in APQ-S dimension scores among sociodemographic subgroups using independent samples *t*-tests and correlations. Those who were older, single or widowed, and/or less educated and/or those with lower monthly net incomes were expected to have more negative perceptions of aging (i.e., higher *timeline*, *consequence negative*, and *emotional representations* scores and lower *consequence positive* and *control* scores). These expectations were based on findings from previous studies [11, 12]. Listwise deletion was used to account for missing sociodemographic data (*n*_single/widow_ = 2; *n*_low education_ = 3; *n*_low income_ = 57).
6. To roughly examine whether Turkish migrants had more negative aging perceptions, subscale means and SDs were calculated and compared with those obtained previously with a general population of Dutch elders residing in Rotterdam [12]. The significance of differences was examined using independent samples *t*-tests.

## Results

### Data screening

Inspection of histograms and Q-Q plots, combined with inspection of means and SDs, revealed skewing of several items. No notable floor or ceiling effect was found (Table 2). Item non-response was distributed evenly and fell below the cut-off point of 10%. Approximately 88% of respondents responded to all items.

**Table 2:** Item characteristics and standardized factor loadings of the APQ-S

Items	Missing (%)	*M*	SD	Med	λ
*Timeline chronic*					
1. I am always aware of my age	3.7	3.97	1.10	4.00	.66
2. I always classify myself as old	4.8	3.35	1.26	4.00	.67
3. I am always aware of the fact that I am getting older	3.7	3.86	1.06	4.00	.85
*Timeline cyclical*					
19. I go through cycles in which my experience of aging gets better and worse	5.3	3.42	1.08	4.00	.50
20. I go through phases of feeling old	4.3	3.85	1.00	4.00	.70
21. My awareness of getting older changes a great deal from day to day	4.6	3.39	1.07	4.00	.42
*Consequence positive*					
4. As I get older I get wiser	4.6	3.30	1.21	4.00	.80
5. As I get older I continue to grow as a person	4.6	3.46	1.13	4.00	.89
6. As I get older I appreciate things more	4.6	3.77	1.11	4.00	.64
*Consequence negative*					
10. Getting older restricts the things that I can do	4.1	3.89	1.01	4.00	.80
11. Getting older makes everything a lot harder for me	4.6	3.87	1.09	4.00	.85
12. As I get older I can take part in fewer activities	4.3	3.95	1.02	4.00	.72
*Emotional representations*					
16. I get depressed when I think about getting older	4.6	3.09	1.30	3.00	.82
17. I worry about the effects that getting older may have on my relationships with others	4.6	3.21	1.23	4.00	.76
18. I feel angry when I think about getting older	4.8	2.52	1.31	2.00	.64
*Control positive*					
7. The quality of my social life in later years depends on me	4.3	3.50	1.12	4.00	.84
8. Whether I continue living life to the full depends on me	4.6	3.45	1.17	4.00	.90
9. Whether getting older has positive sides to it depends on me	4.1	3.51	1.15	4.00	.81
*Control negative*					
13. Slowing down with age is not something I can control	4.8	2.14	1.01	2.00	.76
14. How mobile I am in later life is not up to me	4.3	2.15	.99	2.00	.89
15. I have no control over whether I lose vitality or zest for life as I age	3.9	2.29	1.06	2.00	.71

*Note*. Items on the control negative dimension were recoded so that higher scores are indicative of more perceived control over negative aspects of aging

### Construct validity and reliability

#### Confirmatory factor analysis

**Fig. 1:**
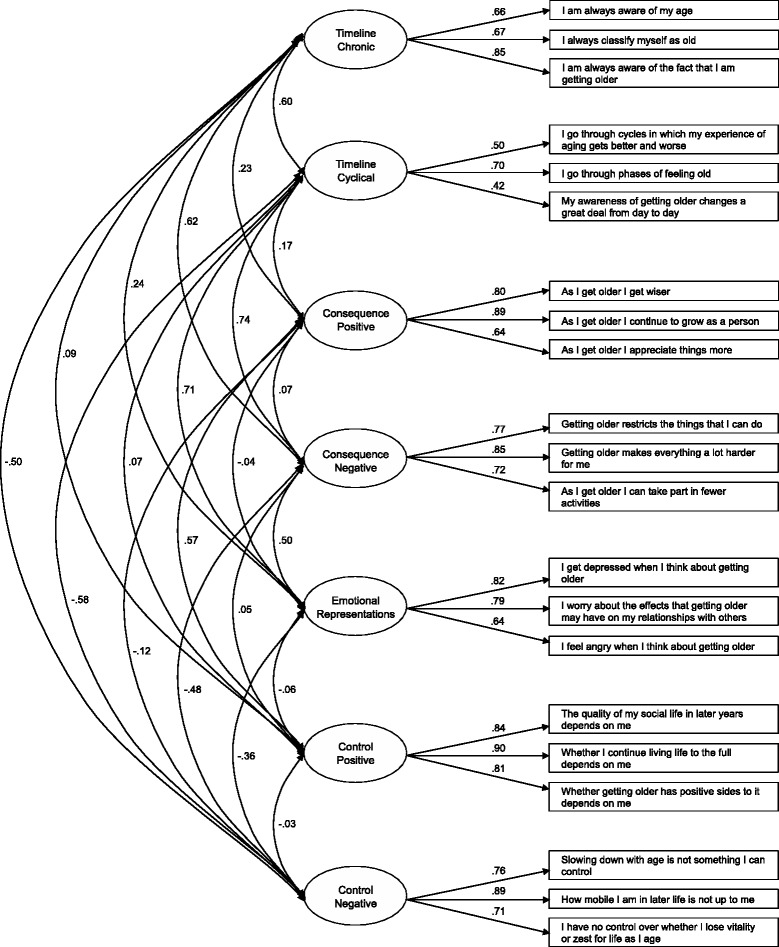
The standardized measurement model

As multiple items violated the assumption of normality, maximum likelihood estimation with robust standard errors and chi-squared statistics was used to analyze the variance-covariance matrix [48, 49, 55]. The resulting latent factor model was found to have an acceptable fit, with RMSEA, SRMR, and CFI statistics within the boundaries of good fit (RMSEA = .06; SRMR = .07; CFI = .90). A large and significant chi-squared value was found (*χ* ^2^ [168] = 453.01, *p* < .001), as expected given the sample size [49]. These results were acceptable and largely comparable to those found for the Dutch APQ-S data (RMSEA = .05; SRMR = .04; CFI = .94; *χ* ^2^[168] = 589.80, *p* < .001) [12] (see Fig. 1 for the standardized measurement model of the Turkish APQ-S). MIs of 10.51–36.51 further revealed several areas of moderate localized strain, with the highest MI indicating potential cross-loading of the *control negative* item “I have no control over whether I lose vitality or zest for life as I age” onto *emotional representations*. However, model adjustment according to this MI did not markedly improve fit or make substantial theoretical sense. Finally, each indicator loaded significantly on its corresponding latent factor and had a standardized factor loading > .40 (Table 2), supporting the convergent validity of the Turkish APQ-S dimensions [49, 53]. Thus, the latent variables appear to have been measured well by their respective indicators. In sum, the hypothesized factor structure of the APQ-S adequately reflects the observed data.

#### Inter-factor correlations

Several significant correlations were found between APQ-S (sub)scales (Table 3). For example, respondents who had more chronic awareness of aging also perceived that they were less in control of negative experiences related to aging, harbored more negative emotions toward aging, and attributed more negative consequences to the aging process. The strongest correlation was found between *emotional representations* and *timeline cyclical*, indicating that those whose aging generated more negative emotional responses also reported more variations in their awareness of the process. However, the correlation did not exceed .80; thus, discriminant validity was not in question [49]. Comparison of the inter-factor correlations with correlations reported by Slotman and colleagues [12] revealed no difference in directionality, further supporting the factor structure of the Turkish APQ-S.

**Table 3:** Turkish and Dutch^a^ APQ-S inter-factor correlations, means and standard deviations, and Turkish APQ-S reliabilities

APQ Dimensions	1.	2.	3.	4.	5.	6.	*M* (SD)	α
*1. Timeline chronic*								
Turkish APQ-S	-						3.73 (.93)^***^	.75
Dutch APQ-S	-						3.35 (.92)^***^	
*2. Timeline cyclical*								
Turkish APQ-S	.31^***^	-					3.55 (.77)^***^	.56
Dutch APQ-S	.44^***^	-					2.84 (.88)^***^	
*3. Consequence positive*								
Turkish APQ-S	.09	.09	-				3.50 (.98)	.80
Dutch APQ-S	.21^***^	.09^**^	-				3.40 (.76)	
*4. Consequence negative*								
Turkish APQ-S	.47^***^	.41^***^	.00	-			3.90 (.90)^***^	.82
Dutch APQ-S	.50^***^	.54^***^	.12^***^	-			3.38 (.83)^***^	
*5. Emotional representations*								
Turkish APQ-S	.21^***^	.54^***^	−.08	.38^***^	-		2.95 (1.07)^***^	.79
Dutch APQ-S	.33^***^	.66^***^	.07^*^	.46^***^	-		2.46 (.85)^***^	
*6. Control positive*								
Turkish APQ-S	−.01	.04	.52^***^	−.02	−.08	-	3.49 (1.03)^***^	.88
Dutch APQ-S	.04	−.06^*^	.27^***^	.01	−.06	-	3.71 (.70)^***^	
*7. Control negative*								
Turkish APQ-S	−.42^***^	−.36^***^	−.05	−.39^***^	−.32^***^	.01	2.19 (.88)^***^	.81
Dutch APQ-S	−.37^***^	−.32^***^	−.17^***^	−.47^***^	−.33^***^	−.12^***^	2.43 (.77)^***^	

*Note*. ^*^ *p* ≤ .05; ^**^ *p* ≤ .01; ^***^ *p* ≤ .001 (two-tailed). Higher means are indicative of higher levels on the respective aging dimensions, e.g., stronger cyclical awareness and more perceived control over positive and negative aspects of aging. APQ-S mean differences between the Turkish and general Dutch population samples were calculated using independent samples *t*-tests. ^a^ Dutch APQ-S refers to the results found in the APQ-S development and validation study, among a general sample of elderly, residing in Rotterdam, the Netherlands (Slotman et al., 2015)

#### Internal consistency

The majority of APQ-S (sub)scales had adequate internal consistency, with Cronbach’s alpha values ranging from .75 (*timeline chronic*) to .88 (*control positive*; Table 3). However, the *timeline cyclical* subscale had a low Cronbach’s alpha value (*α* = .56), indicating questionable internal consistency.

#### APQ-S dimensions and health outcomes

As expected, APQ-S dimensions correlated significantly with well-being and health measures (Table 4). Higher *timeline chronic* and *cyclical*, *consequence negative*, and *emotional representations* scores were associated with worse well-being and health scores. Hence, respondents who were more chronically or cyclically aware of their aging reported lower levels of mental HRQoL and more chronic conditions. Attributing more negative consequences and having more negative emotional responses to aging were also associated with worse physical HRQoL.

**Table 4:** Correlations between the APQ-S dimensions and well-being and health outcomes and socio-demographic subgroup differences

	Timeline chronic	Timeline cyclical	Consequence positive	Consequence negative	Emotional representations	Control positive	Control negative
*Well-being*	−.09	−.14^**^	.32^***^	−.25^***^	−.26^***^	.41^***^	.02
*Physical HRQoL*	−.13^**^	−.09	.07	−.20^***^	−.18^***^	.11^*^	.09
*Mental HRQoL*	−.22^***^	−.20***	.10*	−.27^***^	−.28^***^	.16^**^	.13^*^
*Nr of chronic conditions*	.26^***^	.24^***^	−.21^***^	.38^***^	.26^***^	−.29^***^	−.24^***^
*Age*	.09	.04	−.14^**^	.13^**^	.07	−.16^**^	−.03
*Gender*							
Female	3.88 (.94) ^**^	3.58 (.77)	3.45 (1.03)	4.02 (.93) ^*^	3.00 (1.09)	3.36 (1.10) ^*^	2.16 (.90)
Male	3.61 (.91) ^**^	3.52 (.77)	3.54 (.94)	3.80 (.87) ^*^	2.90 (1.06)	3.59 (.96) ^*^	2.22 (.86)
*Income*							
*<* €1350	3.75 (.88)	3.54 (.79)	3.50 (.96)	3.93 (.86)	2.92 (1.07)	3.51 (.99)	2.20 (.85)
≥ €1350	3.57 (1.07)	3.53 (.76)	3.51 (1.05)	3.70 (1.06)	2.98 (1.02)	3.48 (1.18)	2.07 (.91)
*Education*							
< Elementary schooling	3.88 (.90) ^***^	3.57 (.76)	3.47 (.98)	4.06 (.81) ^***^	3.00 (1.10)	3.42 (1.04)	2.14 (.88)
≥ Elementary schooling	3.55 (.93) ^***^	3.53 (.77)	3.56 (.98)	3.72 (.95) ^***^	2.89 (1.03)	3.58 (1.06)	2.24 (.85)
*Marital status*							
Single/widowed	3.90 (.88) ^*^	3.59 (.75)	3.48 (1.04)	4.13 (.86) ^**^	3.14 (1.13) ^*^	3.31 (1.09) ^*^	2.10 (.87)
Married/living together	3.66 (.95) ^*^	3.53 (.78)	3.52 (.96)	3.81 (.90) ^**^	2.86 (1.04) ^*^	3.56 (1.00) ^*^	2.23 (.87)

*Note*. ^*^ *p* ≤ .05; ^**^ *p* ≤ .01; ^***^ *p* ≤ .001 (two-tailed). Subgroup differences were calculated using independent samples *t*-tests.

Better well-being and health scores were found among respondents with higher *consequence positive, control positive*, and *control negative* scores. For example, those who believed that aging impacts one’s life in a positive manner reported higher levels of well-being (*r =* .32; *p* ≤ .001) and mental HRQoL (*r =* .10; *p* ≤ .05) and fewer chronic conditions (*r = -*.21; *p* ≤ .01). Feeling more control over positive aspects of aging was correlated with better physical HRQoL (*r =* .11; *p* ≤ .05). The strongest correlation was found between the *control positive* score and well-being (*r =* .41; *p* ≤ .001).

#### APQ-S dimensions and sociodemographic variables

Aging perceptions varied according to age, gender, educational level, and marital status (Table 4). Respondents who were female, less educated, and/or single/widowed were more chronically aware of their aging and believed more strongly that aging would negatively affect their lives. Less educated and single/widowed respondents also harbored more negative emotions toward their aging and felt less control over positive aspects of aging. Finally, those who were older believed that they had less control over positive aspects of aging. Furthermore, they attributed fewer positive and more negative consequences to aging. Contrary to expectations, no significant difference was found according to income.

#### Mean differences between Turkish and Dutch APQ-S subscale responses

Several mean APQ-S subscale scores differed significantly between the Turkish migrant sample and the general Rotterdam population sample (Table 3) [12]. Elderly Turkish migrants were more chronically and cyclically aware of their aging. Furthermore, they attributed more negative consequences to and felt more negative emotions toward and less control over their aging. On average, elderly Turkish migrants attributed more positive consequences to aging than did elderly Dutch residents of Rotterdam, but this difference was not significant.

## Discussion

With millions of Turkish migrants and other groups of former guest workers reaching retirement age in countries to which they are not native, examination of how one’s migrant or ethnic minority status and the socioeconomic disadvantages it often implies may shape the experience and perception of old age has become increasingly important. This question becomes especially relevant in light of ample studies that have underscored the negative impacts of maladaptive aging perceptions on well-being and health [11, 13, 26]. To study aging perceptions among elderly migrants, instruments that can be used validly and reliably across ethnic groups are needed. The current study examined the psychometric properties of the APQ-S with a group of community-dwelling elderly Turkish migrants in the Netherlands. It also roughly examined ethnic differences in aging perceptions between these migrants and a general sample of elders residing in Rotterdam.

### Construct validity and reliability

In general, the study results supported the construct validity and reliability of the Turkish-language APQ-S. All model fit statistics were within the boundaries of good fit, with the exception of the chi-squared statistic, which was expected [49]. Furthermore, each item loaded significantly onto its corresponding latent dimension and no large area of localized strain within the model was detected. Inter-factor correlations also supported the construct validity of the Turkish APQ-S in terms of its hypothesized factor structure. Additional evidence for construct validity was found in the significant associations between Turkish APQ-S dimension scores and measures of well-being, health, and sociodemographic characteristics, which were largely in congruence with expectations based on previous studies [11, 12]. Finally, the majority of the APQ-S dimensions were found to have acceptable reliability. The Cronbach’s alpha value for the *timeline cyclical* subscale, however, was notably lower. This result may be due to the difficulty of grasping the meaning of subscale items such as “going through phases or cycles” of feeling old, especially among those with low educational levels (the majority of respondents). It may also indicate that cyclical awareness of aging is not a culturally relevant concept for Turkish elders. Hence, it may not correspond to the way in which this population conceives of the aging process and experiences old age. Qualitative studies may shed further light on this issue.

### Aging perceptions among elderly Turkish migrants

The results of this study provide further support for the possible health merits of positive perceptions and health risks of negative perceptions of aging [13, 15, 19], and provide initial evidence for the its generalizability to Turkish elderly migrants. For example, they suggest that being highly aware of one’s aging, whether chronically or sporadically, may pose a health risk, whereas increased feelings of control over aging may be beneficial to the well-being and health of elderly Turkish migrants. These findings are in line with those of previous studies showing similar associations among native elderly populations [12, 14].

Furthermore, the study results suggest that aging perceptions are shaped by sociodemographic characteristics, such as age, gender, educational level, and marital status, with a possible increased risk for maladaptive aging perceptions among women and people who are older, less educated, widowed, and/or single. Whereas other studies have demonstrated significant associations between income level and perceptions of aging [11, 12, 56], no such association was observed in the current study. This finding, however may be the result of a lack of variance due to the large number of respondents with lower income levels (68.01% had net monthly incomes < €1350).

Finally, this study underscores the importance of studying aging perceptions and their associations with health in this specific elderly group, as Turkish migrants generally had higher scores for negative APQ-S dimensions (i.e., *timeline chronic* and *cyclical*, *consequence negative*, and *emotional representations*) and lower scores for positive dimensions (i.e., *control positive* and *negative*), compared with the general sample of elderly Rotterdam residents [12]. This finding is remarkable, especially given the considerably younger age of the Turkish population (65+ years vs. 70+ years for the Rotterdam sample). Hence, it suggests the existence of ethnic differences in aging perceptions, with elderly Turkish migrants having more negative conceptions of aging and, subsequently, increased health risks. Ethnic differences in aging perceptions may partially explain the disparities in well-being and health found previously between elderly native and Turkish migrant populations [4–6, 10].

Whether these possible ethnic differences are the result of cultural differences in age-related beliefs or differences in socioeconomic resources is an interesting question for future research. For example, the lower levels of perceived control over aging among Turkish elders may be explained by the tendency of people from Islamic societies to ascribe control to outward forces, such as fate or the will of God. This concept is captured in the commonly used Islamic saying *Insh’allah* (‘if god wills’) [57]. Löckenhoff and colleagues [58] also pointed to the existence of multiple cultural-level indicators of aging perceptions in their comparison of age-related beliefs in 26 countries.

The more socially disadvantaged position of elderly Turkish migrants may also have negatively affected their aging perceptions. As the majority of these elders have received little or no schooling and have accumulated few financial resources throughout their lives, many lack sufficient financial means to support themselves in old age, and/or lack knowledge concerning the rights and procedures of services and amenities for elderly individuals. This notion is supported by Steverink and colleagues’ [11] finding of a negative relation between the availability of resources, including education and income, and maladaptive aging perceptions.

### Limitations

Several limitations of this study should be addressed. First, due to the cross-sectional nature of the data, inferences concerning the directionality of the observed associations could not be made. Hence, while more negative and less positive aging perceptions may result in lower levels of well-being and worse health, the opposite may also be the case. Congruent with the latter, scholars have theorized that physical health and well-being are important resources for effective coping with old age, and thus that they affect the personal experience of aging [11, 59, 60]. Second, as data from the first data collection period were used in this study, the preliminary response rate was low. Most non-response was due to the inability to reach respondents after a minimum of two contact attempts, potentially resulting in non-response bias. Accordingly, non-response analyses were conducted. No significant difference in gender was found between respondents and non-respondents. The mean age of these groups, however, differed significantly; on average, respondents were younger than non-respondents (72.11 [SD = 5.10] vs. 72.73 [SD = 5.00], respectively). This difference may be an indication of a selective non-response. However, the low preliminary response rate was expected, as contact and response rates are generally low among ethnic minorities [61]. In a large-scale study of ethnic-minority elders in the Netherlands, Turkish elders were found to have the lowest response rate [4]. As in the current study, non-response was due mainly to the failure to contact a large number of respondents within the data collection period in that study. Hence, non-response may be reduced by increasing the number of contact attempts and lengthening the period of data collection [61, 62]. Third, a large amount (13%) of data on income was missing, which may explain the lack of a significant association with the seven APQ-S dimensions in this study. Fourth, test-retest reliability was not assessed in this study. Further research is needed to assess the instrument’s stability and reliability over time. Fifth, more research is needed to understand why dimensions such as *timeline chronic* and *control negative* were not associated significantly with well-being. Finally, we did not include a pre-test of the instrument among a panel of experts to ensure content validity. Based on the results and interviews, however, we do not have any indication there is a problem with the content.

## Conclusion

As elderly Turkish migrants are among the most disadvantaged elderly groups in society, studies focusing on factors related to their health and well-being are vital to ensure their healthy aging. Specific attention should be paid to age-related perceptions, which have been found to be of key importance to the health and well-being of elderly people and have been suggested to be useful targets in interventions. Hence, given its sound psychometric properties in terms of reliability and construct validity, the Turkish-language APQ-S may prove to be an invaluable tool for future gerontological studies.

## Additional file

Additional file 1: Aging Perceptions Questionnaire Short (APQ-S) perceptions of aging scale (English original [12, 14] and Turkish translation). (DOCX 20 kb)

## Abbreviations

APQ-S: Aging perceptions questionnaire short
CCMO: Dutch central committee on research involving human subjects
CFA: Confirmatory factor analysis
CFI: Comparative fit index
HRQoL: Health-related quality of life
MCS: Mental component summary scale
MI: Modification indices
PCS: Physical component summary scale
RSMEA: Root mean square error of approximation
SEM: Structural equation modelling
SPF-IL: Social production function instrument for the level of wellbeing
SRMR: Standardized root mean square residual
WMO: Medical research involving human subjects act
