# Supplementary material for: Validation of the Aging Perceptions Questionnaire Short on a sample of community-dwelling Turkish elderly migrants
Source: Health Qual Life Outcomes. 2017 Feb 21;15:42. doi: 10.1186/s12955-017-0619-7 (PMC5320659; doi:10.1186/s12955-017-0619-7)
Supplement: Additional file 1: — Aging Perceptions Questionnaire Short (APQ-S) perceptions of aging scale (English original [12, 14] and Turkish translation). (DOCX 20 kb) [file 12955_2017_619_MOESM1_ESM.docx]

**Additional file:** Aging Perceptions Questionnaire Short (APQ-S) perceptions of aging scale (English original [12,14] and Turkish translation).

| 1. I am always aware of my age | Her zaman yaşımın farkındayım |
| --- | --- |
| 1. I always classify myself as old | Kendimi her zaman yaşlı biri olarak görüyorum |
| 1. I am always aware of the fact that I am getting older | Her zaman yaşlandığımın farkındayım |
| 4. As I get older I get wiser | Ne kadar yaşlanıyorsam, o kadar daha akıllı oluyorum |
| 5. As I get older I continue to grow as a person | Ne kadar yaşlanıyorsam, kişiliğim de o kadar gelişiyor |
| 6. As I get older I appreciate things more | Yaşlandıkça her şeye daha çok değer veriyorum |
| 7. The quality of my social life in later years depends on me | Hayatımın ileri kalan yaşlarında sosyal hayatımın kalitesi tamamıyla bana bağlıdır |
| 8. Whether I continue living life to the full depends on me | Her zaman dolu bir hayat yaşayıp yaşamayacağım tamamen bana bağlıdır |
| 9. Whether getting older has positive sides to it depends on me | Yaşlılığın da olumlu taraflarının olup olmadığı tamamıyla bana bağlıdır |
| 10. Getting older restricts the things that I can do | Yaşlanmak imkanlarımı sınırlamaktadır |
| 11. Getting older makes everything a lot harder for me | Yaşlanmak her şeyi benim için daha zor hale getirmektedir |
| 12. As I get older I can take part in fewer activities | Yaşlandıkça faaliyetlere daha az katılabiliyorum |
| 13. Slowing down with age is not something I can control | Daha ileri yaşlarda ağır hareket etmek benim elimde olan bir şey değildir |
| 14. How mobile I am in later life is not up to me | Daha ileri yaşlarda ne kadar hareketli olacağım hiçbir şekilde benim elimde değildir |
| 15. I have no control over whether I lose vitality or zest for life as I age | Daha ileride daha az canlı olursam veya yaşam zevkimi kaybedersem, bununla ilgili hiçbir şey yapamam |
| 16. I get depressed when I think about getting older | Yaşlanmayı düşünmek beni karamsar yapıyor |
| 17. I worry about the effects that getting older may have on my relationships with others | Yaşlanmanın benim başkaları ile olan ilişkilerime yapabileceği olası etkileri düşündüğümde endişeleniyorum |
| 18. I feel angry when I think about getting older | Yaşlanmayı düşünmek beni kızdırıyor |
| 19. I go through cycles in which my experience of ageing gets better and worse | Yaşlılıkla ilgili olarak bazen daha iyi, bazen de daha kötü deneyimlerimin olduğu bir döngüde yer alıyorum |
| 20. I go through phases of feeling old | Kendimi yaşlı hissettiğim zamanlar oluyor |
| 21. My awareness of getting older changes a great deal from day to day | Yaşlanmak hakkındaki bilincim günden güne değişiyor |
